# Increasing uptake of NHS Health Checks: a randomised controlled trial using GP computer prompts

**DOI:** 10.3399/BJGP.2020.0887

**Published:** 2021-07-13

**Authors:** Natalie Gold, Karen Tan, Joseph Sherlock, Robin Watson, Tim Chadborn

**Affiliations:** Public Health England Behavioural Insights, London, UK; visiting professor in practice, Centre for Philosophy of Natural and Social Sciences, London School of Economics and Political Science, London, UK.; Public Health England Behavioural Insights, London, UK; director of innovation and program development, Vera Institute of Justice, New York, US.; London, UK; senior behavioral researcher, Center for Advanced Hindsight, Duke University, Durham, US.; Public Health England Behavioural Insights, London, UK; PhD student, Department of Anthropology, Durham University, Durham, UK.; Public Health England Behavioural Insights, London, UK.

**Keywords:** cardiovascular disease, computer systems, general practice, primary health care, primary prevention

## Abstract

**Background:**

Public Health England wants to increase the uptake of the NHS Health Check (NHSHC), a cardiovascular disease prevention programme. Most invitations are sent by letter, but opportunistic invitations may be issued and verbal invitations have a higher rate of uptake. Prompting staff to issue opportunistic invitations might increase uptake.

**Aim:**

To assess the effect on uptake of automated prompts to clinical staff to invite patients to NHSHC, delivered via primary care computer systems.

**Design and setting:**

Pseudo-randomised controlled trial of patients eligible for the NHSHC attending GP practices in Southwark, London.

**Method:**

Eligible patients were allocated into one of two conditions, (a) Prompt and (b) No Prompt, to clinical staff. The primary outcome was attendance at an NHSHC.

**Results:**

Fifteen of 43 (34.88%) practices in Southwark were recruited; 7564 patients were eligible for an NHSHC, 3778 (49.95%) in the control and 3786 (50.05%) in the intervention. Attendance in the intervention arm was 454 (12.09%) compared with 280 (7.41%) in the control group, a total increase of 4.58% (OR = 2.28; 95% CI = 1.46 to 3.55; *P*<0.001). Regressions found an interaction between intervention and sex (OR = 0.65; 95% CI = 0.44 to 0.86, *P* = 0.004), with the intervention primarily effective on males. Comparing the probabilities of attendance for each age category across intervention and control suggests that the intervention was primarily effective for younger patients.

**Conclusion:**

Prompts on computer systems in general practice were effective at improving the uptake of the NHSHC, especially for males and younger patients.

## INTRODUCTION

The NHS Health Check (NHSHC) programme is a population-level intervention to prevent cardiovascular disease (CVD) events. It targets all adults in England aged 40–74 years who do not have a pre-existing CVD condition.^1^ According to the regulations, eligible patients should be invited to an NHSHC every 5 years. As well as allowing for early CVD diagnosis, patients who do not have CVD but who present with elevated CVD risk are offered behaviour-change support related to risk factors including obesity, smoking, and alcohol consumption. However, between the start of the programme in 2014/2015 until 2018/2019, uptake only averaged 48.1%.^2^ There is an opportunity to improve the current programme’s impact by increasing the number of eligible people having an NHSHC.

The statutory duty for implementation of the NHSHC programme lies with local authorities, who are permitted flexibility regarding details of programme delivery, including the method by which patients are invited to the NHSHC.^1^ The most common invitation method is a letter, which also has a patient information leaflet enclosed.^3^^–^4 However, written invitations may be complemented with opportunistic invitations that are offered when patients have contact with GP surgery staff for some other reason. An observational cohort study in Stoke-on-Trent showed that the odds of a patient attending an NHSHC were almost three times higher when they were given a verbal invitation (telephone or face-toface) either alone or in combination with the letter, compared with those invited by letter only, even when controlling for other predictors.^5^ Another observational study in Luton showed that face-to-face invitations had an overall uptake rate of 71.9% and telephone invitations had an uptake of 43.0%, compared with 29.5% for letter invitations.^6^

Therefore, one way to increase uptake would be to increase the number of opportunistic verbal invitations that are issued. Evidence suggests that computerised prompts may be an effective way of doing that. Systematic reviews show that point-of-care computerised prompts for physicians have been successful at improving adherence to processes of care^7^^,^^8^ and medications management,^9^ and several randomised controlled trials show that they can increase the ordering of tests or preventive therapies for patients in hospital.^10^^–^12 There is also evidence from systematic reviews that prompts are most effective when they are provided automatically (rather than practitioners having to activate the system) and occur at the point of decision.^13^^–^14

**Table:** How this fits in

Most NHS Health Check (NHSHC) invitations are sent by letter, but verbal opportunistic face-to-face invitations have a higher rate of uptake. Point-of-care prompts to healthcare staff, delivered via computer systems, have been successful at improving adherence to processes of care and medications management, and increasing the ordering of tests or preventive therapies for patients in hospital. This pseudo-randomised controlled trial shows that prompts to clinical staff in primary practice to invite patients to their NHSHC, delivered via practice computer systems, can increase uptake. The prompts were particularly effective at increasing uptake among males and younger age groups, who are usually less likely to attend.

This study’s primary aim was to assess the effect on uptake of automated prompts to clinical staff to invite patients to NHSHCs, delivered via primary care computer systems.

## METHOD

### Participant recruitment and randomisation

This was a pseudo-randomised controlled trial with a parallel design, where patients were allocated in roughly equal numbers to the intervention and the control, based on their ages. The study took place in the London Borough of Southwark and was implemented from 2 May 2015 to 17 July 2015, which was the period that the information technology (IT) prompts were active.

The estimated sample size was 1774 individuals in each condition, with a total sample of 3548. This was calculated in Stata version 13.1 based on the following parameters: power of 0.8; significance level of 0.05; baseline uptake of 10%, using the 2014/2015 uptake in Southwark as a baseline and then increasing it for a conservative estimate; a 5-week study period; and a percentage increase as a result of the intervention of 3%, which is a conservative estimate based on previous literature. Then, all the eligible patients in the practices who agreed to participate were randomised. Once launched, practical constraints around study duration lifted and it was decided that the intervention would be active for a 12-week period; this enabled a larger sample size of 7816.

The randomisation was a pseudo-randomised process based on the ages of the participants. The participants with quinquennial ages (ending in 0 or 5) were stratified and systematically split. The reason for this was that letter invitations are sent to people in Southwark when they reach one of these milestones, so these were the patients who were likely to have already received a letter invitation in 2015 and most likely to respond to a prompt. The practices were also divided into two areas and stratified based on this, in order to account for differing levels of deprivation. Fully random allocation was not possible within the restrictions of the IT system, so the researchers devised an allocation sequence to assign patients to groups that would be as free from bias as possible (see Supplementary File 1 for details).

### Intervention

There were two conditions: (a) Prompt (intervention) and (b) No Prompt (control). The intervention was a prompt to clinical staff, ‘Patient due NHS Health Check’, which appeared on the screen when the file was opened. The full list of clinical staff who may have received the prompt is in Box 1. If the member of staff moved the cursor over the prompt, then they were given the instruction, ‘Please offer the patient an appointment for their free NHS Health Check’. A list of the actions that would trigger the receipt of the prompt by clinical staff is in Box 1.

**Box 1.** A list of the clinical staff who could access the system and the actions that would trigger the receipt of the prompt by clinical staff

**Clinical Staff**	**Triggers**
Dispenser	Add a consultation
Counsellor	Book appointment
Clinical assistant	Load patient record
Clinical team manager	Register a patient
Therapist	Save consultation
Medical technical officer	Update patient record
Health professional	
Health care student	
Midwife	
Midwife manager	
Nurse manager	
Social worker	
Technician — healthcare scientist	
Technician — PS&T	
Student technician	
Clinical practitioner	
Staff nurse	
Nurse practitioner	
Nurse	

*PS&T = precision solutions and telematics.*

Installation of the IT protocol for the prompt was offered to all GP practices in Southwark. Fifteen out of 43 practices agreed to have the protocol installed on their computer system and participate in the trial. Only patients eligible for an NHSHC (aged 40–74 years, who did not have a pre-existing CVD condition and had not previously had an NHSHC in the last 5 years) at the participating GP practices were included in the study. All eligible patients were randomised.

The new template for the prompts were developed for EMIS Web and installed by the clinical commissioning group (CCG) in the participating practices. The data that were extracted from the clinical system contained no patient identifiable information. Once data had been extracted, the same algorithm that was used to determine the groups for the prompts was used to create the groups for analysis.

### Outcome measures

The primary outcome was attendance at an NHSHC. Data on attendance of the NHSHC were stored on EMIS software and were extracted by Southwark CCG. Data were automatically extracted from patient records, meaning there was no outcome assessor as such, and blinding was not necessary. Extraction of data continued until 28 August 2015 to allow a further 6 weeks for patients to attend an NHSHC once the intervention had ended. Individuals who had a birthday and changed age during this study were excluded from the sample that was extracted because the change in age interfered with the randomisation algorithm (see Supplementary File 1 for details). In order to minimise the impact of this exclusion on the sample size, twelve separate extractions were run — one for each week the trial was in field — which were merged into one data file for analysis.

The primary outcome was recorded as a binary variable (0 = Did not attend, 1 = Attended). There were no secondary outcomes in this study. Ethnicity was grouped according to NHS Data Dictionary codes.^15^

### Statistical analysis

A χ^2^ test of independence was used to check the distribution of demographics across intervention and control, and a multilevel logistic regression was used for the primary outcome. Three models were fitted and reported below. The first is an unadjusted model that contains only the intervention as a fixed effect (Model 1). The second adds practice as a random effect and the demographics as fixed effects (Model 2). The final model added an interaction between sex and the intervention, and allows varying slopes for the different GP practices, to investigate differences in effectiveness of the intervention depending on sex and on the individual GP practice (Model 3). Interactions with age or ethnicity were not included, since the multiple levels of those variables would have made interaction effects too difficult to interpret, but predicted probabilities of attendance (the model’s prediction that someone with these characteristics would attend) were generated for the demographic groups for each level of the intervention (intervention and control), holding all other variables constant. Note that these predictions were generated from a model that excludes the random effects, meaning the pooled intercept for practice is used in the predictions. The analysis was conducted in R version 3.6.3.

## RESULTS

**Figure 1.**
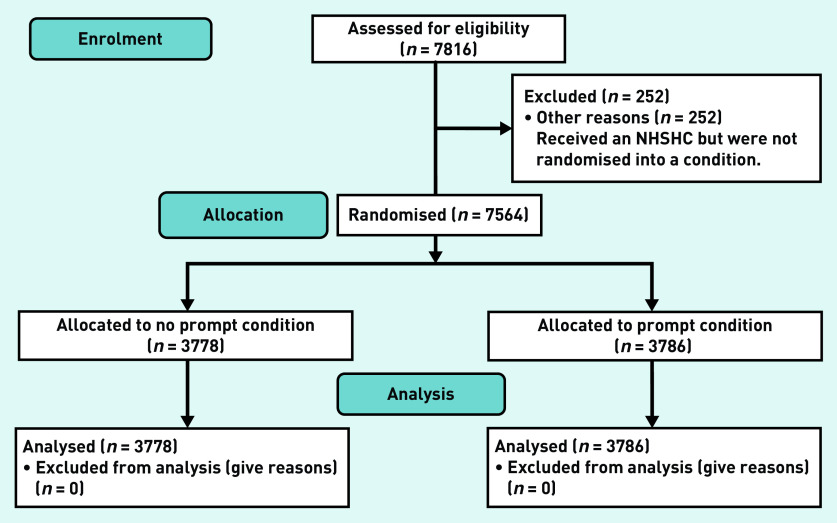
*Trial flowchart. NHSHC = NHS Health Check.*

The data used in this analysis comprise 7816 individuals who were registered in a participating practice and eligible for an NHSHC between the dates of 2 May 2015 and 17 July 2015, the pre-specified start and end dates of the trial. Of these, 252 (3.22%) were not randomised into a condition for the trial and were excluded from the analysis at this stage, owing to technical issues in the practices when the randomisation was rolled out. See Figure 1 for the trial flowchart. This left 7564 complete cases, with 3778 (49.95%) in the control and 3786 (50.05%) in the intervention. See Table 1 for the uptake figures, broken down by practice. There were 15 different GP practices with a median of 381 patients and an interquartile range of 282.5. Two practices did not offer any health checks; it is not known why.

**Table 1.** Total number of patients and counts and percentages of those who attended an NHSHC across GP practices by trial condition

**GP practice**	**Patients (Control) *N***	**Patients who attended health check (Control) *N* (%)**	**Patients (Intervention) *N***	**Patients who attended health check (Intervention) *N* (%)**
1	163	9 (5.52)	208	45 (21.63)
2	160	9 (5.63)	221	38 (17.12)
3	190	0 (0)	160	0 (0)
4	173	25 (14.45)	128	18 (14.06)
5	116	34 (29.31)	153	51 (33.33)
6	443	42 (9.48)	356	18 (5.06)
7	198	8 (4.04)	223	26 (11.66)
8	167	23 (13.77)	125	15 (12.00)
9	241	21 (8.71)	299	65 (21.74)
10	614	31 (5.05)	468	24 (5.13)
11	277	13 (4.69)	368	29 (7.88)
12	446	41 (9.19)	518	62 (11.96)
13	182	0 (0)	122	0 (0)
14	153	7 (4.58)	163	14 (8.59)
15	255	17 (6.67)	274	49 (17.88)
Aggregate	3778	280 (7.41)	3786	454 (11.99)

*NHSHC = NHS Health Check.*

The baseline demographics of the intervention and control group showed no statistically significant differences for sex and ethnicity, but there was a relationship between age and the intervention, χ^2^ (6, *n* = 7564) = 17.983, *P*<0.01 (see Table 2 for the complete set of tests). Inspecting the distributions further, it seems that there is an overrepresentation of the 50–54 years category in the control group (20.57% of that condition) compared with the intervention group (17.51%), which may explain this result.

**Table 2.** Counts and percentages of demographic variables across the intervention and control groups, along with results from a χ^2^ test of independence

		**Control *N*= 3778**	**Intervention *N*= 3786**	χ**^2^ (df)**	***P-*value**
Sex *N* (%)	Male	1608 (42.56)	1541 (40.70)	2.6152 (1)	0.106
	Female	2170 (57.44)	2245 (59.30)		

Age category, years *N* (%)	40–44	1090 (28.85)	1129 (29.82)	17.983 (6)	0.006^a^
	45–49	892 (23.61)	956 (25.25)		
	50–54	777 (20.57)	663 (17.51)		
	55–59	488 (12.92)	461 (12.18)		
	60–64	260 (6.88)	287 (7.58)		
	65–69	180 (4.76)	171 (4.52)		
	70–74	91 (2.4)	119 (3.14)		

Ethnicity *N* (%)	White	1814 (48.01)	1740 (45.96)	8.1694 (5)	0.147
	Black	891 (23.58)	934 (24.67)		
	Asian	135 (3.57)	140 (3.70)		
	Unknown	501 (13.26)	488 (12.89)		
	Mixed	117 (3.10)	106 (2.80)		
	Other	320 (8.47)	378 (9.98)		

*df = degrees of freedom.*

The number of participants attending an NHSHC increased from 280 (7.41%, *n* = 3778) in the control to 454 (11.99%, *n* = 3786) in the intervention group, χ^2^ (1, *n* = 7564) = 44.753, *P*<0.001. Compared with the control, this is a 4.58% absolute increase in uptake and a 61.81% relative increase.

The unadjusted model (Model 1, Table 3) showed that people in the intervention group had higher odds of attending an NHSHC (odds ratio [OR] = 1.70; 95% confidence intervals [CI] = 1.46 to 1.99, *P*<0.001). The effect of the intervention remains significant, even after adjusting for demographic variables and variance between practices in Model 2 (OR = 1.62; 95% CI = 1.37 to 1.90, *P*<0.001), and it even increased in Model 3 (OR = 2.62; 95% CI = 1.46 to 3.55, *P*<0.001).

**Table 3.** Logistic regressions of uptake of an NHSHC estimating the effect of the intervention, with control letter as a baseline; adjusted model includes sex (reference category male), age (reference category 40–44 years), and ethnicity (reference category white) as covariates and GP practice as a random effect. *P*-values for random effects are log likelihood tests between models that include and omit the random effect

	**Model 1^a^: (Pseudo R^2^= 0.006)**		**Model 2^b^: (Pseudo R^2^ =0.408)**		**Model 3^c^: Pseudo R^2^= 0.410)**	
	**OR (CI)**	***P-*value**	**OR (CI)**	***P-*value**	**OR (CI)**	***P-*value**
Intercept	0.08 (0.07 to 0.09)	<0.001	0.10 (0.05 to 0.19)	<0.001	0.07 (0.03 to 0.15)	<0.001
Intervention	1.70 (1.46 to 1.99)	<0.001	1.62 (1.37 to 1.90)	<0.001	2.28 (1.46 to 3.55)	<0.001
Female	—	—	0.99 (0.84 to 1.16)	0.86	1.32 (1.02 to 1.71)	0.036
45–49 years	—	—	0.70 (0.57 to 0.86)	<0.001	0.69 (0.56 to 0.85)	<0.001
50–54 years	—	—	0.71 (0.57 to 0.90)	0.0047	0.78 (0.62 to 1.00)	0.04501
55–59 years	—	—	0.71 (0.55 to 0.93)	0.0132	0.77 (0.59 to 1.01)	0.055
60–64 years	—	—	0.55 (0.38 to 0.79)	0.0011	0.55 (0.38 to 0.78)	<0.001
65–69 years	—	—	0.64 (0.42 to 0.98)	0.0395	0.64 (0.42 to 0.98)	0.0418
70–74 years	—	—	0.61 (0.37 to 1.03)	0.06	0.65 (0.39 to 1.10)	0.110
Black	—	—	0.93 (0.77 to 1.12)	0.44	0.92 (0.76 to 1.12)	0.405
Asian	—	—	0.75 (0.49 to 1.14)	0.18	0.74 (0.49 to 1.13)	0.161
Mixed	—	—	1.35 (0.90 to 2.03)	0.14	1.37 (0.92 to 2.06)	0.125
Other	—	—	0.64 (0.48 to 0.86)	0.0031	0.64 (0.48 to 0.86)	0.0032
Unknown	—	—	0.13 (0.08 to 0.22)	<0.001	0.13 (0.08 to 0.22)	<0.001
Intervention^a^ Female	—	—	—	—	0.62 (0.44 to 0.86)	0.004495
Variation in intercept	—	—	1.27	<0.001	1.31	<0.001
between practices, SD						
Variation in slopes for the	—	—	—	—	0.56	<0.001
intervention between practices, SD						

a *Model 1: an unadjusted model that contains only the intervention as a fixed effect.*

b *Model 2: adds practice as a random effect and the demographics as fixed effects.*

c *Model 3: an interaction between sex and the intervention is added, and as well allowing varying slopes for the different GP practices, to investigate differences in effectiveness of the intervention depending on sex and on the individual GP practice. CI = confidence interval. NHSHC = NHS Health Check. OR = odds ratio. SD = standard deviation.*

**Figure 2.**
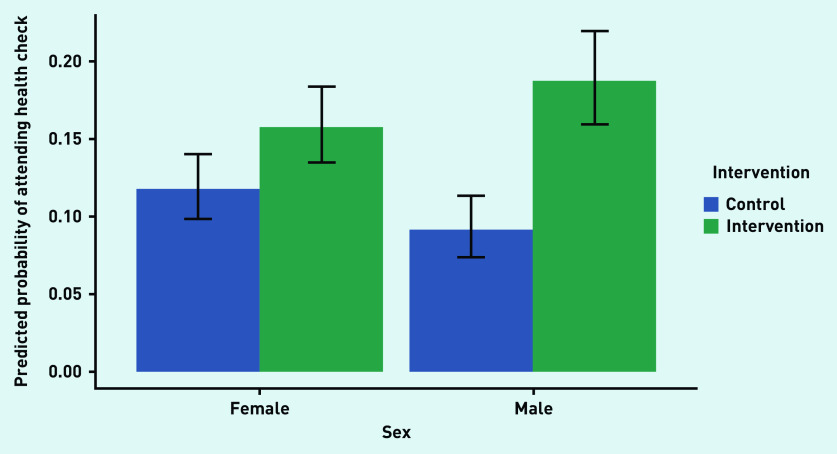
*Predicted probability (95% CI) of males and females attending an NHSHC in the intervention and control groups.* *NHSHC = NHS Health Check.*

There was no statistically significant effect of sex in the model that included demographics but not interaction effects (Model 2, reference category male) (OR = 0.99; 95% CI = 0.84 to 1.16, *P*<0.86). However, when the interaction of the intervention and sex was included (Model 3), not only a differential effect of the intervention on sex was found, with a main effect of sex (OR = 1.32; 95% CI = 1.018 to 1.710, *P* = 0.036), but also a significant interaction effect (OR = 0.62; 95% CI = 0.44 to 0.86, *P* = 0.0045). Inspecting the predicted probabilities of attendance for males and females for each level of the intervention (Figure 2) suggests that the intervention was primarily effective for males.

**Figure 3.**
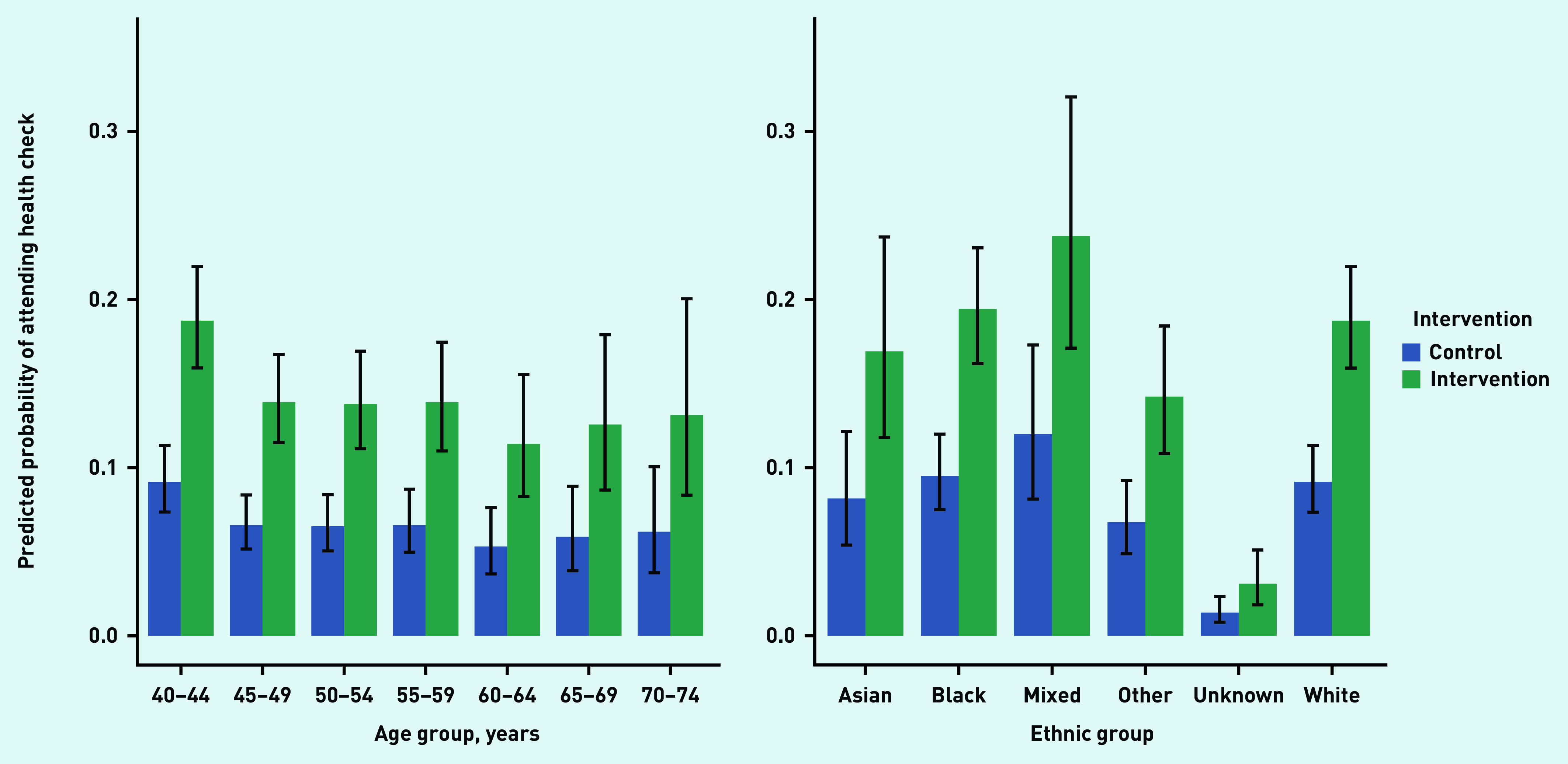
*Predicted probabilities for all categories of age and ethnicity to attend an NHSHC in the intervention and control groups. NHSHC = NHS Health Check.*

The youngest age group, aged 40–44 years, was most likely to attend an NHSHC, with all older age groups being less likely to attend an NHSHC than the baseline group. In Model 2, with the reference category for age being 40–44 years, ORs were <1 for all groups and *P*<0.05 for all ages except 70–74. Model 3 follows a similar pattern, except for the 55–59 years age bracket, which was now *P =* 0.055. See Table 3 for the full model. Predicted probabilities of attendance for each age category across intervention and control are shown in Figure 3, and these suggest that the intervention was primarily effective for younger age groups.

Unknown (OR = 0.13; 95% CI = 0.08 to 0.22, *P*<0.001) and Other (OR = 0.64; 95% CI = 0.48 to 0.86, *P* = 0.003) ethnicities were less likely to attend an NHSHC than the baseline category of white (from Model 2, with the same pattern of results in Model 3; see Table 3 for the full models). The predicted probabilities of attendance for each ethnic group across intervention and control do not show any clear patterns (Figure 3).

There was a significant variation between intercepts across practices: standard deviation (SD) = 1.27 (Model 2), SD = 1.31 (Model 3), χ^2^ (1, *n* = 7564) = 250.076, *P*<0.001 for both models. This shows that there were differences in uptake across practices. When the slope of the regression line was allowed to vary for each practice (Model 3) so the differential effect of the intervention on the different practices could be seen, the practice’s slopes varied significantly across practices and were negatively correlated with the intercepts (*r* = −0.24), χ^2^ (2, *n* = 7564) = 21.573, *P*<0.001. This suggests that the intervention had less of an effect in practices that already had higher uptake of NHSHCs.

## DISCUSSION

### Summary

This study found that prompts to clinical staff, delivered by practice computer systems, was associated with an increased uptake of the NHSHC: 4.58% higher than the control group in absolute terms. There was an interaction effect of the intervention and sex, with the intervention being more effective for males. The predicted probabilities of attendance also suggested that the intervention was more effective for younger age groups. There was variation in uptake by GP practice and the intervention was more effective in practices that had a lower baseline rate of uptake.

### Strengths and limitations

The study had several strengths. The intention to treat method captures the effects of the intervention as it will be practised, including potential non-adherence to protocol, and does not require monitoring of compliance. Another strength was pseudo-randomisation, which means that the characteristics of the intervention and control groups should be balanced, allowing the authors to make a causal inference that the intervention caused the difference in uptake between the two groups. The study also used clearly defined exclusion criteria based on NHSHC guidance, and had a relatively large sample size.

One limitation is that there could have been a selection bias, since only 15 (34.9%) out of 43 practices in Southwark consented to have the prompts installed on their IT systems. This may have had an impact on generalisability of the study, as practices that did not consent to the prompts may be less likely to engage with the prompts and invite patients than practices who agreed to participate in the study.

There could also have been some contamination between arms, since patients in the same practice were randomised to different arms. Because of the modest number of practices that were available in Southwark, a cluster-randomised design was not used; randomising at the level of the clinician was impracticable and would not have eliminated contamination. Being prompted to invite some patients to NHSHCs could have made practice staff more likely to remember to invite others. In that case, NHSHC uptake might have improved in the control group as well as in the intervention. That implies that the results are a conservative estimate of the increase in uptake that can be achieved by installing computer-based prompts.

Southwark is an urban location with a lower proportion of white patients than is typical across the country and a higher proportion aged <65,^16^ which might affect the generalisability of the findings, especially because the intervention was more effective for younger patients. Furthermore, the uptake of the NHSHC was low across all practices included in this trial, which might make the prompt more effective than in a higher-uptake environment. Uptake varied between practices; practice variation is usual for the NHSHC,^17^^–^20 since practices may organise the delivery differently, and this is taken into account in the models.

The study also had a relatively short duration. There is some concern that prompts may be annoying for the clinical staff and may result in ‘prompt fatigue’.^21^ That is, over time, staff may engage less with the prompts as they become routine and less novel, especially if there is a high frequency of alerts. If clinical staff are desensitised to prompts, then they may become less effective over time. However, a previous study that took place over a 5-year period found that prompting still remained effective after 5 years.^22^

The study’s primary outcome measure was uptake of the NHSHC, not improvement in health outcomes. However, evaluation of the NHSHC programme has found that it has decreased cardiovascular events,^23^ and there is no evidence of inequity by ethnicity and deprivation.^24^ This evidence highlights the potential role of primary care to deliver preventive interventions, which can also save money in the long term.

The trial was intent-to-treat, and there are no data on how or whether particular healthcare professionals acted on the prompts. Therefore, although it can be inferred that the intervention caused the increase in uptake (owing to the randomised design), details such as whether particular clinical staff were more likely to act on the prompts or whether characteristics of the patient influenced the likelihood of prompts being offered are not known.

Clinician prompts, such as the one used in this research, will only influence patients who are already engaging with their local GP services. Therefore, consideration should be given to health equity, as there may be particular groups who are less likely to attend at the GP practice. Verbal telephone invitations have also been effective at improving uptake of the NHSHC,^25^ indicating that they could be used to supplement and to make contact with hard-to-reach patients groups.

### Comparison with existing literature

This pseudo-randomised controlled trial shows that automated prompts delivered via a computer system can be effective at increasing the uptake of the NHSHC, a CVD risk assessment that is part of a prevention programme. This study strengthens the evidence base, since one previous study also found that prompts can increase uptake of preventive procedures (although that study did not employ randomisation).^22^ However, another randomised controlled trial found that neither computer-based nor manual prompts increased adolescent vaccination rates.^26^ There were various salient differences from the present study: it took place in the US instead of the UK, participants were adolescents and not 40–74-year-olds, and the outcome was an immunisation, not a risk assessment. Further research is needed to ascertain the generalisability of this study’s results. However, prompts are very low-cost interventions: after installation there are no financial costs associated with the prompt itself, although there is the time cost for the clinician to click the prompt and offer appointments. Clearly, not every procedure can have a prompt, so there would have to be prioritisation.

This intervention was more effective on males and younger age groups. National uptake statistics show that females are more likely to attend an NHSHC than males and that older patients are more likely to attend than younger ones.^18^^,^^20^^,^^23^^,^^27^ Previous trials using behaviourally informed invitation letters have also found that females and older patients are more likely to attend,^19^^,^^20^ and a trial of telephone invitations did not find differential effects of the invitation method by demographic group.^25^ In contrast, the present study did not find differential overall uptake for sex, and when the interaction between sex and intervention was entered into the model, it was found that the intervention was more effective on males. For age, it was found that the youngest age group, those aged 40–44 years, were most likely to attend, and it was also found that the intervention was most effective for younger age groups. This suggests that verbal invitations might be more effective than letter invitations at reaching groups who generally have a low attendance.

### Implications for research and practice

Computer-based prompts delivered to staff in primary care were effective at improving the uptake of the NHSHC, and seemed especially effective at increasing uptake in groups that are usually least likely to attend, such as males and younger patients. Further research could determine whether their effectiveness generalises to increasing the uptake of other preventive procedures, such as vaccinations.
